# Nanoparticle translocation and multi-organ toxicity: A particularly small problem

**DOI:** 10.1016/j.nantod.2019.03.010

**Published:** 2019-06

**Authors:** Jennifer B. Raftis, Mark R. Miller

**Affiliations:** University/BHF Centre for Cardiovascular Science, University of Edinburgh, Edinburgh, United Kingdom

**Keywords:** Nanoparticle, Nanomaterials, Air pollution, Translocation, Systemic actions, Multi-organ toxicity

## Abstract

Environmental nanoparticles and manufactured nanoparticles (MNMs) can share many of the same physicochemical properties and, therefore, could have similar toxicological profiles. Inhalation of nanoparticles in air pollution has effects throughout the body; however, the potential for inhaled MNMs to affect multiple organs requires further investigation. The biological mechanisms that link nanoparticles deposition in the lung to their systemic actions remain to be established; however, the passage of nanoparticles into the blood (“translocation”) represents a compelling explanation. This article highlights experimental work in animals and man showing that inhaled gold nanoparticles pass into the blood and accumulate at sites of vascular disease. The article discusses the properties of nanoparticles that could influence translocation and highlights some avenues for future research. The processes described have clear relevance, both for MNMs and sources of nanoparticles in air pollution. The authors emphasise the need for risk assessment of potential nanoparticle exposure routes that consider the multiple organ systems.

## Highlights

• Environmental and manufactured nanoparticles share physicochemical properties and could have similar toxicological profiles.
• Particles in air pollution have effects on many organ systems. Is the same true for manufactured nanoparticles?
• Recently inhaled nanoparticles were shown to pass into the circulation in man and amass at sites of vascular inflammation.
• Translocation of nanoparticles into systemic circulation could underlie their toxicity to multiple organs.
• The authors stress the importance of risk assessment for manufactured nanoparticles that considers multiple organ systems.

## Graphical abstract

**Figure:**
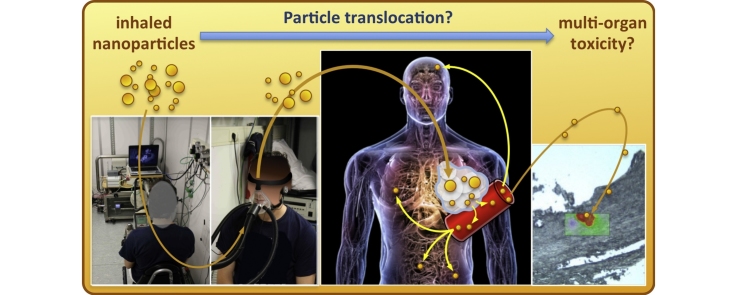

Over the last few decades, there has been increasing recognition of the shared interests of researchers that investigate the health effects of ultrafine particulate matter (PM) in air pollution and the toxicological effects of engineered nanoparticles/manufactured nanomaterials (MNM) [1]. Both types of substance have the defining property of their small size (ultrafine particles are particles with diameter less than 100 nm; nanomaterials have at least one dimension smaller than 100 nm) and both classes of particles share some of the physicochemical properties that are believed to be critical to their biological actions and toxicity [2]. However, there are also clear parallels between the methods used to research these fields and the challenges that researchers face in assessing their potential health effects. While the health effects of particulate air pollution have been recognised for many decades (even centuries), ‘nanotoxicology’ is a relatively young area of research. However, the rapid development of nanotechnologies for a vast array of applications (including human administration for medical diagnostics and drug delivery) underscores the importance of nanotoxicological research. While the annual number of publications for nanotechnology now overshadows air pollution research [1], the health effects of particles in air pollution are well established by comparison.

Globally, air pollution has been estimated to be responsible for several million premature deaths every single year [3]. While air pollution is a complex cocktail of different chemical constituents from many different sources, particulate matter (PM) is believed to be the key species driving the adverse cardiovascular (which are responsible for two-thirds of the attributable mortality [3]) [4]. These associations are more robust for PM_2.5_ than PM_10_ (PM with diameters less than 2.5 and 10 μm respectively) [5]. Ultrafine PM (PM with a diameter less 100 nm; “PM_0.1_”; i.e. nanoparticles) are likely to have an even greater biological action on an equivalent mass basis. Due to current limitations in measuring ultrafine PM in the environment, studies can rarely ascribe health parameters to ultrafine alone. Nonetheless, PM_10_ and PM_2.5_ metrics will include a proportion of ultrafine particles (especially where combustion sources are present), and these particles can be present in large numbers with a large reactive surface area, even where the contribution to mass metrics is low. As we will discuss, the nanosize of ultrafine PM also brings with it the possibility of additional means by which these particles can affect health.

The pulmonary effects of PM exposure are well established, and the last two decades have cemented the cardiovascular system as an organ system that is acutely vulnerable to PM in air pollution. Controlled exposure studies to pollutants such as diesel exhaust (that is rich in ultrafine particles) can induce lung inflammation [6] and impairment of cardiovascular parameters [4,7]. However, there is now a burgeoning list of other extrapulmonary effects associated with exposure to air pollution (albeit PM_10_, PM_2.5_, or proximity to roadways, rather than ultrafine PM *per se*). These include diabetes and metabolic syndrome [8], neurological effects (impaired cognition, depression, Alzheimer’s disease and Parkinson’s disease [9,10]) and potential teratogenic and epigenetic effects from exposure *in utero* [11,12]. There are also emerging reports of air pollution affecting the kidneys [13], the gastrointestinal system [14], the liver [15], stem cells [16], mortality following organ transplantation [17], diseases of the skin [18], fertility [19] and various cancers [[20], [21], [22], [23]] (Fig. 1).

**Fig. 1:**
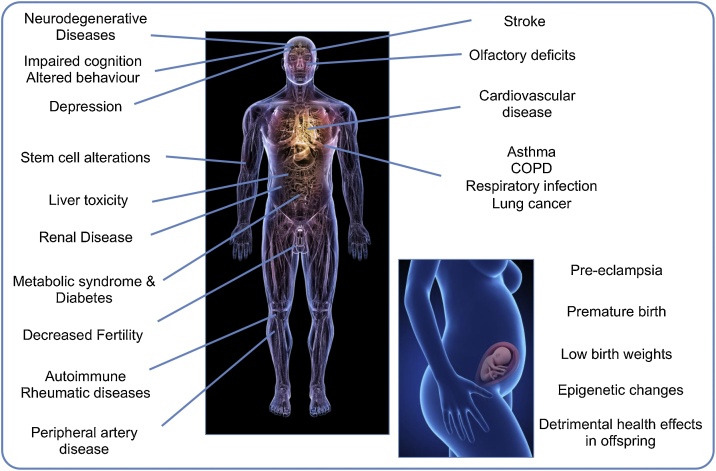
Particulate air pollution has been linked to a wide range of conditions affecting many different organs of the body.

The biological mechanisms that account for the extra-pulmonary effects of inhaled PM remain unknown. Of particular interest are the mechanisms by which effects on the lung progress to other organs. Identifying these pathways is fundamental for air pollution research and nanotoxicology. Inhalation remains a likely route of accidental exposure to nanomaterials (e.g. in an occupational or environmental setting), yet investigations into extrapulmonary effects tend to focus on basic blood biochemistry and gross pathology, rather than physiological function in specific organ systems. Three central pathways linking exposure in the lung to effects on distant organs have been theorised: 1) induction of lung inflammation that signals to the circulation 2) activation of alveolar sensory receptors leading to alterations in neurological function and changes in endocrine release, and 3) passage of smaller nanoparticles into the circulation (“translocation”) where they directly harm organs [24].

There is now a consistent body of evidence from animal studies which demonstrate nanoparticles can cross the alveolar barrier and settle in extrapulmonary organs. Until recently, empirical confirmation of a similar process in humans has been lacking. Nemmar et al. showed the passage of technetium particles into the blood following inhalation in healthy volunteers [25]. However, subsequent groups failed to reproduce these experiments [26] and suggested that leaching of the radiolabel from the particle could account for the earlier findings. In 2017, through a collaboration with scientists in the Netherlands, our group demonstrated translocation of inhaled gold nanoparticles using human subjects [27] (Fig. 2). We used high-resolution (limit of quantification: 0.03 ng gold/g of blood) inductively coupled plasma mass spectrometry to detect total gold in blood and urine of healthy volunteers following a 2-h inhalation of 5 nm gold nanoparticles (primary size). Surprisingly gold was still present in blood and urine of the volunteers three months after exposure, at levels higher than the initial 24-h period, pointing towards systemic retention and delayed urinary excretion. These observations align with a recent study in rats using 20 nm radiolabelled gold, showing that initial urinary clearance is slow and that blood levels of gold were higher at 28 days after exposure compared to earlier time-points [28]. We went on to demonstrate that translocation was size-dependent, with greater translocation of smaller nanoparticles into the blood (<30 nm) and urine (<10 nm) (primary particle sizes).

**Fig. 2:**
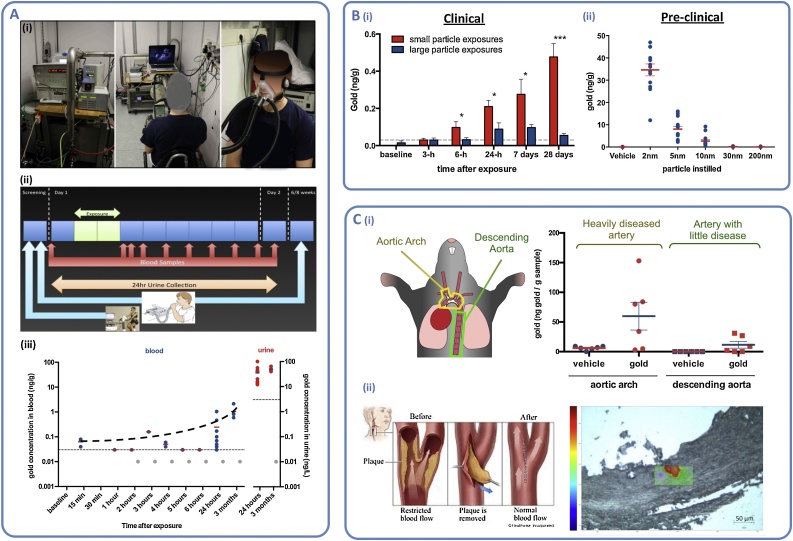
Translocation of inhaled gold nanoparticles in mice and human subjects (data from Miller & Raftis et al. 2017) [26] A. (i) Inhalation of gold nanoparticles (5 nm primary particle size) in healthy volunteers during moderate exercise; (ii) Study protocol; (iii) Detection of gold in blood and urine by high-resolution inductively coupled plasma mass spectrometry, following 2-h inhalation of gold nanoparticles (thin horizontal dotted lines represent limit of quantification (LOQ), grey symbols are below LOQ but above limit of detection). B. Small nanoparticles translocated more readily than large particles in (i) healthy human volunteers (following inhalation of primary particle sizes: ∼5 nm and ∼30 nm) and (ii) mice (∼24-h following instillation of suspensions). C. Accumulation of nanoparticles in atherosclerotic blood vessels; (i) Preferential nanoparticle accumulation in heavily diseased arteries of apolipoprotein-E knockout mice; (ii) Use of Raman confocal microscopy to detect gold in the plaques taken from the carotid arteries of patients with a history of stroke, following inhalation of gold nanoparticles the preceding day.

Despite using a relatively high dose of gold (∼0.4 mg total inhaled dose) and a very high sensitivity detection method, the level of gold in blood was close to the limit of detection (0.03 ng/g) at time points <24-h from the end of the exposure [29]. The question then remains, is the translocation of these low amounts of nanoparticles physiologically relevant? This is a challenging question to answer. We chose gold nanoparticles for a variety of practical reasons, the most important being that they would not cause harm to the volunteers and could be reliably detected. Controlled exposures of dilute diesel exhaust (a pollutant rich in combustion-derived nanoparticles) caused marked cardiovascular actions in human subjects, including impairment of blood vessel relaxation, promotion of thrombosis and increased the susceptibility of the heart to ischemia [4]. These alterations were shown to be driven by the particulate component of diesel exhaust [30,31], although these investigations were not able to address specifically if particle translocation to the cardiovascular system accounted for these effects.

To address this matter, we investigated the possible fate of translocated nanoparticles once in the blood [27]. While the mass dose of translocated particles in the blood is low, particle numbers could be sufficient to cause disproportional biological effects if they reached areas of the body that are especially susceptible to their effects. Using an animal model of atherosclerosis (a disease characterised by the build-up of fats and lipids in the arteries than can occlude blood flow) we demonstrated that 5 nm gold nanoparticles instilled to the lung could be detected in vascular tissue 24-h later. Importantly, the accumulation of gold was greater in diseased arteries compared with disease-free vessels. As a final study, we demonstrated that inhaled gold nanoparticles could be detected in atherosclerotic lesions of the carotid arteries in human subjects with a history of ischemic stroke.

These results ‘add another piece to the jigsaw’ as to how inhaled nanoparticles mediate their systemic effects. The findings are intriguing and raise many questions. From a practical perspective, we now need to confirm if these observations hold for particles other than gold, in particular to environmental nanoparticles such as diesel exhaust particles (DEP). Combustion-derived nanoparticles have a vast array of harmful chemical species on their surface, which contribute to its oxidative capacity, and pro-inflammatory potential [24]. These properties make it highly likely that if DEP reaches inflamed tissues in the same way as gold nanoparticles do, it will be more likely to promote disease (especially so with cumulative exposure over a lifetime) and potentiate rupture of atherosclerotic plaques (the trigger that instigates events such as a heart attack or stroke). Whether or not engineered nanoparticles would promote disease, in the same way, is far from clear. However, free radical generation, induction of cellular oxidative stress and pro-inflammatory responses are hallmarks of nanotoxicity [2]. Therefore, many would state there is a reasonable case for limiting exposure to nanomaterials with similarly low size ranges until these concerns are addressed.

A limitation of our study is that we did not address what biological and particle characteristics determine translocation. Other groups have shown in animal models that both particle size (and, by the same token, surface area) and charge are important determinants to translocation [[32], [33], [34]]. Interestingly, the coating on the surface of the particle from either particle opsonisation [35] or intentional surface modification [36] also affects translocation processes, while the age of the (healthy) animal does not [28]. These particle properties (and others) may also affect the time-course of translocation and clearance mechanisms [37,38]. The inflammatory status of the lung may also play a role in translocation through changes in alveolar permeability. Research suggests that certain sub-populations (e.g. the young, elderly or those with pre-existing disease) could be more susceptible to the adverse effects of air pollution [39]; is the same true for manufactured nanomaterials and could this influence the potential applications for these materials?

Translocation of nanoparticles has far-reaching implications for the fields of air pollution research and nanotoxicology. While our focus has been chiefly from a cardiovascular perspective, translocation potentially explains how inhaled particles mediate effects throughout the body. It raises concerns that other nanoparticles could have similar insidious effects. If translocated nanoparticles selectively localise in areas of inflammation, then what are the implications for other inflammatory conditions or chronic inflammation associated with various cancers? If nanoparticles can cross the alveolar barrier, what other barriers can they cross? The blood-brain-barrier, or the placenta? Indeed, the brain and maternal-foetal effects are hot topics in air pollution research [40,41], and of interest (or concern) for nanomedicine e.g. for imaging, drug delivery or as a therapeutic agent? [[42], [43], [44]]. If nanoparticles can cross these barriers in significant numbers, then what are the dose implications for nanomaterials designed for injection directly into the bloodstream? Could manufactured nanomaterials act as carriers for other chemicals in a manner analogous to diesel exhaust particles carrying chemicals from incomplete combustion on their surface? While we are at risk of descending into a Cassandra complex, there is little doubt that the fate of nanoparticles in the body is paramount to understanding their health risks. A better understanding of how nanoparticles cross between organs will greater assist the development of new theragnostic agents. With the accumulated knowledge from both air pollution research and nanotoxicology, there is the potential to predict and avoid any probable toxicity of nanomaterials; ultimately allowing us to harness their unique properties safely, to enhance our lives.

## Conflicts of interest

The authors declare that they have no conflicts of interest.
